# Periosteal Flaps Enhance Prefabricated Engineered Bone Reparative Potential

**DOI:** 10.1177/00220345211037247

**Published:** 2021-09-11

**Authors:** A.G. Abu-Shahba, T. Wilkman, R. Kornilov, M. Adam, K.M. Salla, J. Lindén, A.K. Lappalainen, R. Björkstrand, R. Seppänen-Kaijansinkko, B. Mannerström

**Affiliations:** 1 Department of Oral and Maxillofacial Diseases, University of Helsinki and Helsinki University Hospital, Helsinki, Finland; 2 Department of Oral and Maxillofacial Surgery, Faculty of Dentistry, Tanta University, Tanta, Egypt; 3 Department of Oral and Maxillofacial Surgery, Helsinki University Hospital, Helsinki, Finland; 4 Department of Equine and Small Animal Medicine, Faculty of Veterinary Medicine, University of Helsinki, Helsinki, Finland; 5 Department of Veterinary Biosciences, Faculty of Veterinary Medicine, University of Helsinki, Helsinki, Finland; 6 Finnish Centre for Laboratory Animal Pathology (FCLAP), HiLIFE, University of Helsinki, Helsinki, Finland; 7 Department of Mechanical Engineering, Aalto University, Espoo, Finland

**Keywords:** bioreactors, flap prefabrication, periosteum, sheep, mandibular reconstruction, vascularization

## Abstract

The clinical translation of bone tissue engineering for reconstructing large bone defects has not advanced without hurdles. The *in vivo* bioreactor (IVB) concept may therefore bridge between bone tissue engineering and reconstructive surgery by employing the patient body for prefabricating new prevascularized tissues. Ideally, IVB should minimize the need for exogenous growth factors/cells. Periosteal tissues are promising for IVB approaches to prefabricate tissue-engineered bone (TEB) flaps. However, the significance of preserving the periosteal vascular supply has not been adequately investigated. This study assessed muscle IVB with and without periosteal/pericranial grafts and flaps for prefabricating TEB flaps to reconstruct mandibular defects in sheep. The sheep (*n* = 14) were allocated into 4 groups: muscle IVB (M group; *n*_M_ = 3), muscle + periosteal graft (MP group; *n*_MP_ = 4), muscle + periosteal flap (MVP group; *n*_MVP_ = 4), and control group (*n*_Control_ = 3). In the first surgery, alloplastic bone blocks were implanted in the brachiocephalic muscle (M) with a periosteal graft (MP) or with a vascularized periosteal flap (MVP). After 9 wk, the prefabricated TEB flaps were transplanted to reconstruct a mandibular angle defect. In the control group, the defects were reconstructed by non-prevascularized bone blocks. Computed tomography (CT) scans were performed after 13 wk and after 23 wk at termination, followed by micro-CT (µCT) and histological analyses. Both CT and µCT analysis revealed enhanced new bone formation and decreased residual biomaterial volume in the MVP group compared with control and MP groups, while the M group showed less new bone formation and more residual biomaterial. The histological analysis showed that most of the newly formed bone emerged from defect edges, but larger areas of new bone islands were found in MP and MVP groups. The MVP group showed enhanced vascularization and higher biomaterial remodeling rates. The periosteal flaps boosted the reconstructive potential of the prefabricated TEB flaps. The regenerative potential of the periosteum was manifested after the transplantation into the mechanically stimulated bony defect microenvironment.

## Introduction

Mandibular defects remain a challenge for reconstruction to achieve predictable aesthetic and functional outcomes. Bone tissue engineering has been expected to achieve a paradigm shift in the reconstructive approaches (Wang et al. 2011; Chanchareonsook et al. 2014). However, it faces critical hurdles related principally to the lack of mature vasculature in large constructs, not to mention the required access to good manufacturing practice facilities and related regulatory licenses (Williams 2019; Mastrullo et al. 2020). The *in vivo* bioreactor (IVB) strategy is a promising translational approach that harnesses the patient’s body to prefabricate vascularized autologous tissues for reconstructive purposes. This approach combines the potentials of conventional reconstructive surgery and bone tissue engineering (Tan et al. 2004; Huang, Kobayashi, et al. 2016).

Prefabricated tissue-engineered bone (TEB) flaps have been explored in preclinical models and several clinical cases with variable outcomes (Huang, Kobayashi, et al. 2016; Kasper et al. 2017; Akar et al. 2018; Tatara et al. 2019). However, most of these studies involved the use of exogenous cells, growth factors, or other harvested cell sources (e.g., autologous bone or bone marrow aspirates). For a better clinical translatability, an ideal IVB technique should leverage the inherent regenerative capacity of the employed tissues to obviate or minimize the use of seeded cells and growth factors. Therefore, different IVB techniques should be assessed for maximizing the balance between bone regeneration and remodeling (Heliotis et al. 2006; Huang, Kobayashi, et al. 2016; Huang, Liu, et al. 2016).

Muscles have been employed as an IVB to enhance ectopic neovascularization and bone regeneration into an incorporated suitable scaffold (Khouri et al. 1991; Ayoub et al. 2007; Mesimäki et al. 2009; Huang, Kobayashi, et al. 2016). Muscle-IVB features a well-vascularized tissue with adequate bulk for later harvesting as a composite TEB flap to reconstruct complex maxillofacial defects (Khouri et al. 1991; Mesimäki et al. 2009). Nevertheless, muscle IVB requires the addition of osteoinductive/osteogenic factors for a predictable heterotopic osteogenesis (Khouri et al. 1991; Spalthoff et al. 2018).

The periosteum is a well-vascularized osteogenic organ (McKibbin 1978; Hutmacher and Sittinger 2003; Malizos and Papatheodorou 2005). Large animal studies have employed the periosteum in IVBs via 2 approaches: the first has successfully used vascularized costal periosteal envelopes, created by the extraction of rib segments, for generating vascularized TEB flaps in sheep and for allograft revitalization in pigs (Runyan et al. 2010, 2014; Tatara et al. 2015, 2019). This approach is promising for augmenting the available rib bone stock; however, it is restricted by the size of the rib periosteum and involves a relative morbidity for rib segments harvesting. The second approach has involved nonvascularized periosteal grafts transplanted into the greater omentum of minipigs (Naujokat et al. 2019, 2020). Although this approach violates the anterior abdominal wall, it has reflected the versatility of transplanting periosteal grafts into a distant IVB site for customized defect-specific bone tissue regeneration. However, both approaches have combined the periosteal tissues with different biomaterials and/or osteoinductive/osteogenic factors, thus complicating the elucidation of the intrinsic potential of the periosteal tissues and the role of its vascularity.

This study aimed at investigating the effects of preserving the vascular supply of periosteal flaps as compared to nonvascularized transplanted periosteal grafts for prefabricating engineered myoosseous flaps. We assessed muscle IVB with and without periosteum for prefabricating TEB flaps employing alloplastic bone blocks with no additional cell-source or osteoinductive agents. Furthermore, the functional performance of the transplanted TEB flaps was investigated for the reconstruction of large mandibular bone defects in sheep.

## Materials and Methods

### Alloplastic Bone Blocks

This study involved a total of 18 purchased SmartBone blocks (15 × 30 × 20 mm) (#NFHU011210; Industrie Biomediche Insubri S/A). These bone blocks (BBs) are biohybrid in nature, consisting of bovine-derived mineral matrix, which is reinforced with resorbable poly(lactic-co-caprolactone) copolymer and RGD-exposing collagen fragments for surface activation (Pertici et al. 2015; Ferracini et al. 2019; Sallent et al. 2020).

### Animals, Surgery, and Study Design

This animal study was approved by the Finnish Animal Experiment Board (ESAVI/16103/2018; August 17, 2018). The surgical procedures were designed based on thorough reviewing of the sheep anatomy and presimulation on a sheep cadaver. The sheep were purchased from a licensed commercial vendor and housed as a flock in group pens under the standard housing conditions in the large animal facility of the Laboratory Animal Centre of the University of Helsinki. The sheep were acclimatized for 4 wk before any intervention. They were closely monitored by veterinarians and trained animal caretakers.

**Figure 1.**
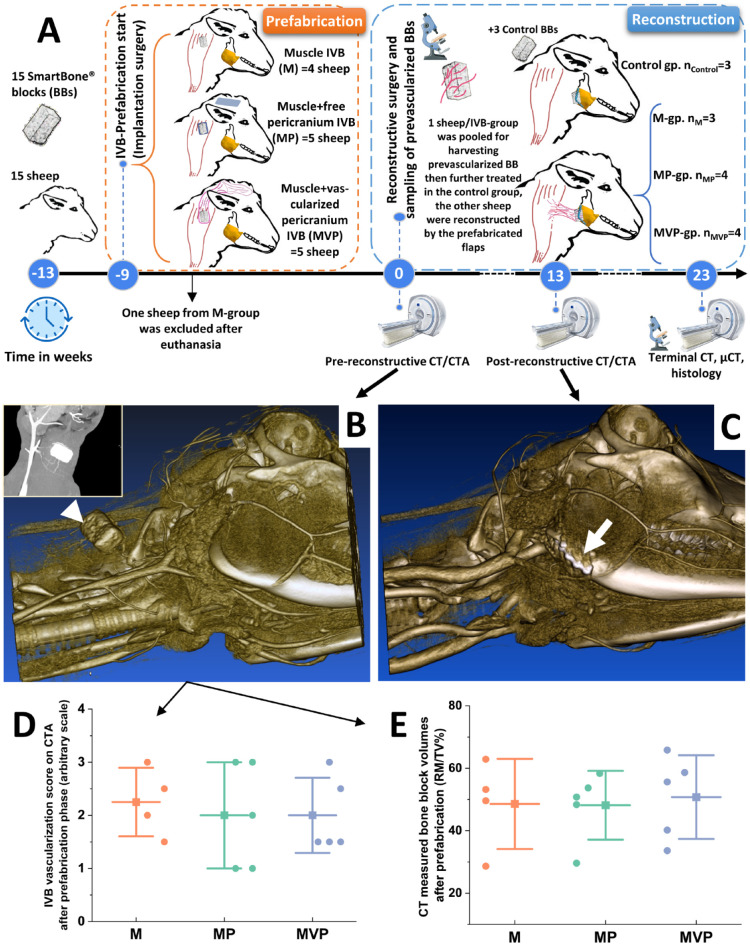
The study design and CT angiography results. The general outline of the study events is presented (A). At the end of the prefabrication phase, all sheep underwent pre-reconstructive computed tomography (CT) scans for assessing the prevascularized bone blocks (BBs) (white arrowhead in B). During the healing after the reconstructive transplantation surgery, the sheep underwent post-reconstructive CT scans for assessing the stability of the transplanted flaps into the mandibular angle defects (white arrow in C) and evaluating the ongoing new bone formation and biomaterial changes. These parameters were further reassessed by terminal-endpoint CT scans. The data of CT angiography (CTA) (exampled by the small window in B) were used for constructing 3-dimensional models (B, C). The analysis of the pre-reconstructive CTA revealed no differences among the employed *in vivo* bioreactors (IVBs) regarding the detected vasculature scaling around the BBs (D). The CT-measured BB volumes did not show major differences among the tested IVB conditions at the end of the prefabrication phase (E). The boxplots show mean (–■–), SD (whiskers), and averaged observation values (•). *n*_M_ = 4. *n*_MP_ = 5. *n*_MVP_ = 5. M, intramuscular pouch; MP, a pericranial nonvascularized graft with the muscular pouch; MVP, pericranial vascularized flap with the muscular pouch.

This study involved 15 skeletally mature female Texel and Crossbred sheep (24–35 mo [26.6 ± 4.3 mo]; 51–65 kg [56.4 ± 4.3 kg]). All sheep underwent 2 surgical procedures under general anesthesia (GA) (Fig. 1A). In the first surgery, BBs were implanted with 3 different IVB techniques (1 block per sheep/IVB). The sheep were randomly allocated into 5 sheep per tested IVB. The tested IVBs comprised an intramuscular pouch (M) in the rostral part of brachiocephalic muscle, a pericranial nonvascularized graft with the muscular pouch (MP), and a pericranial vascularized flap with the muscular pouch (MVP) (Appendix Fig. 1).

After a *prefabrication period* of 8 to 11 wk, the sheep underwent the second surgery (reconstructive phase). The prefabricated TEB flap was raised as a pedicled flap for reconstructing a critical-sized defect (CSD) of the ipsilateral mandibular angle. The surgeon was blinded to the groups in the second surgery. In 3 sheep (1 per each IVB), the prefabricated TEB blocks were harvested for histological evaluation, and the CSDs were reconstructed using fresh alloplastic BBs as a control. The details of the GA and surgical procedures are provided in Appendix.

One sheep from the M group suffered from postoperative cardiopulmonary complications 1 wk after the first surgery; it was euthanized and therefore excluded from the study. The postoperative course for surgeries is provided in the Appendix. In summary, the later assessment of the reconstructive phase comprised 14 sheep (*n* = 14) in 4 groups: the control group (*n*_Control_ = 3), M group (*n*_M_ = 3), MP group (*n*_MP_ = 4), and MVP group (*n*_MVP_ = 4). Subsequent analyses were performed by 2 independent observers, of whom only 1 was blinded due to practical restraints.

### Computed Tomography Analysis

After an average of 9 wk from the first surgery, the sheep underwent a computed tomography (CT) scan of the head and neck under GA, first without (CT) and then with intravenous contrast material (CT angiography [CTA]). This aimed at assessing the vascularization around the prefabricated TEB flaps immediately before transplantation (pre-reconstructive CT) (Fig. 1B). Sheep underwent another CT/CTA scan under GA at an average 13 ± 5 wk after the last surgery (Fig. 1C). The variation in timing of follow-up CT was due to unforeseen regulatory events in the CT facility; however, this variation was equally balanced among groups. Terminal-endpoint CT scan was performed on the heads of the sheep immediately after euthanasia 23 ± 1 wk after the last surgery. All the scans were performed using LightSpeed VCT 64 slice CT Scanner (GE Medical Systems). Details of scanning parameters are in the Appendix.

### Micro-CT Scans

After the terminal CT, the reconstructed CSD with a rim of native bone was excised and fixed in 4% paraformaldehyde (PFA) at 4°C. The micro-CT (µCT) scans were carried out with a GE phoenix nanotom s system (General Electric Sensing and Inspection Technologies/Phoenix X-ray) at the University of Helsinki X-Ray Micro-CT Laboratory. Details of scanning parameters are in the Appendix.

### Analysis of CT and µCT Data

For the visualization of the CT data, JiveX Image Report (VISUS Health IT GmbH) was used. The CTA visualization was performed with 3Diagnosys RealGUIDE 5.0 (3DIEMME Srl) or Horos (Horos Project). The vascularization of the prefabrication sites was scaled employing 4 tiers: 0 = no blood vessels (BVs), 1 = mild (a single BV to 1 direction), 2 = moderate (BVs from 2 directions), and 3 = extensive (several branches around the BB). The volume of the newly formed bone (NB/TV%) and the residual biomaterial (RM/TV%) were assessed at the post-reconstructive CTs and the terminal µCT in CTAnalyser (CTAn) software 1.18.8.0 (Bruker). Details of the analysis are provided in the Appendix.

In a parallel setting, the change in the volume of the BB was evaluated by comparing the 3-dimensional (3D) reconstructed models from CT data sets of each time point to estimate the resorbed volume at the terminal endpoint as compared to the initial pre-reconstruction volume. The detailed protocol is provided in the Appendix.

### Histological Analysis

Samples for histological analysis were collected from the explanted BBs (1 from each IVB group) after the prefabrication phase and from the mandibular samples after the terminal µCT. The samples were fixed in 10% neutral buffered formalin for 12 d and divided into segments to allow the analysis of different areas of the reconstructed defect. Decalcification of the samples was performed in 0.5 M ethylenediaminetetraacetic acid (EDTA) 7.5 pH for 12 wk, followed by routine processing for paraffin embedding, and sectioned at 4 µm thickness. The decalcified sections were stained with hematoxylin and eosin (H&E) and Masson’s trichrome (MTC). Selected sections were stained with picrosirius red, reticulin, and Movat’s pentachrome staining. Sections from the explanted BB samples underwent immunohistochemical (IHC) staining using the anti–von Willebrand factor (vWF) antibody (1:1,000; rabbit polyclonal, Ab6994; Abcam) to assess vascularization. The technique details are provided in the Appendix.

A mid-defect segment from each sheep was processed for undecalcified sections by BioSiteHisto (BioSiteHisto Oy) and stained by Masson Goldner trichrome (MT) stain. Processing details are provided in the Appendix. For the subsequent histological analyses and measurements, the slides were digitalized as a whole-slide image with Pannoramic 250 FLASH II (3DHISTECH) with a 20× air objective, viewed, and analyzed using CaseViewer version 2.4 (3DHISTECH). The areas occupied by the newly formed bone with its marrow spaces and those occupied by the residual biomaterial and fibrovascular stroma were measured.

### Statistical Analysis

The results are presented as means ± standard deviations. Except for the BB samples after prefabrication phase, the averaged technical replicates per sheep were analyzed. Data were analyzed in OriginPro (2020-SR1-9.7.0.188; OriginLab Corporation). The paired samples *t* test was applied to assess differences between the 2 CT time points. The 1-way analysis of variance (ANOVA) was performed with Bonferroni-corrected post hoc tests to analyze specific sample pairs for significant differences. Equality of variances was preassessed by Levene’s test. Details of the statistical analyses are reported in the Appendix. Statistical significance was set at *P* < 0.05.

## Results

### Evaluation of the Prefabrication Phase

**Figure 2.**
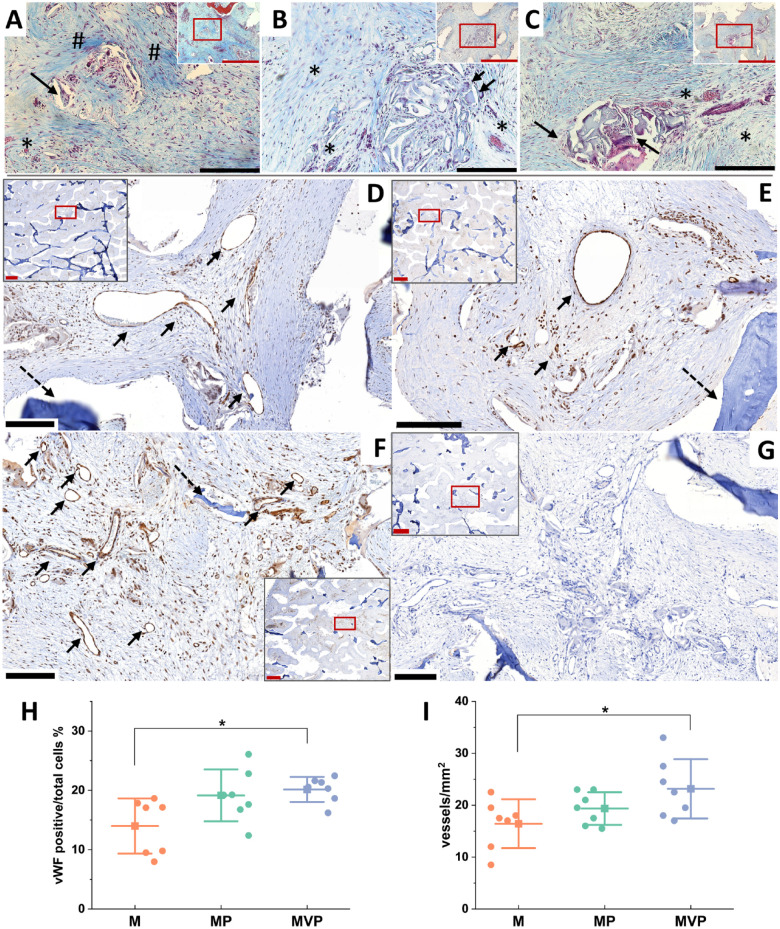
Histological findings after the prefabrication phase. Photomicrographs of the Masson’s trichrome (MTC)–stained sections for the bone blocks (BBs) after the prefabrication phase in the tested *in vivo* bioreactors (IVBs): M (A), MP (B), and MVP (C). No ectopic bone formation was seen within the BBs. Active vascularization and degradation of the biomaterial were evident in all samples with associated macrophages and multinucleated giant cells (MNGCs) (black arrows in A–C). Biomaterial pores were infiltrated by well-vascularized fibrovascular stroma (*). Relatively less degradation and more fibrotic stroma were seen in M samples (# in A). Representations of the immunohistochemistry (IHC) for von Willebrand factor (vWF) and density of blood vessels in the prefabricated tissue-engineered bone (TEB) samples in different IVBs: M (D), MP (E), and MVP (F) after the prefabrication phase. More vascularization was seen in MVP samples, especially when compared to M samples. The black arrows show the detected blood vessels (D–F), and dashed arrows show residual biomaterial. The negative control for IHC is depicted (G). The quantified vWF positive/total cells (%) was higher in MVP samples compared with M samples as was the density of the blood vessels (vessels/mm^2^) (H, I). Red scale bars in section overview = 1,000 µm; in higher magnification (for red boxes), the black scale bars = 200 µm. The boxplots show mean (–■–), SD (whiskers), and averaged measurements from segments of the BB samples (•), *P < 0.05. M, intramuscular pouch; MP, a pericranial nonvascularized graft with the muscular pouch; MVP, pericranial vascularized flap with the muscular pouch.

The pre-reconstructive CTA showed clear vasculature around the implanted blocks in all groups. The scoring of the vascularization from CTA did not show significant differences among the tested IVBs (Fig. 1D). The CT analysis for the volumes of BBs at the end of prefabrication phase revealed nonsignificant difference in their volumes (RM/TV%) (Fig. 1E). During transplantation surgery, the prefabricated TEB flaps showed obvious vascularization of the prefabricated TEBs with bleeding through the biomaterial pores (Appendix Fig. 2C and Appendix Video). Histologically, The MTC-stained sections showed no ectopic new bone formation in any of the IVBs at the end of the prefabrication phase (Fig. 2A–C). The IHC revealed a higher percentage of vWF-positive cells/total cells and increased BV density in MVP group sections (Fig. 2D–I).

### Post-Reconstructive Analyses

#### CT analysis

**Figure 3.**
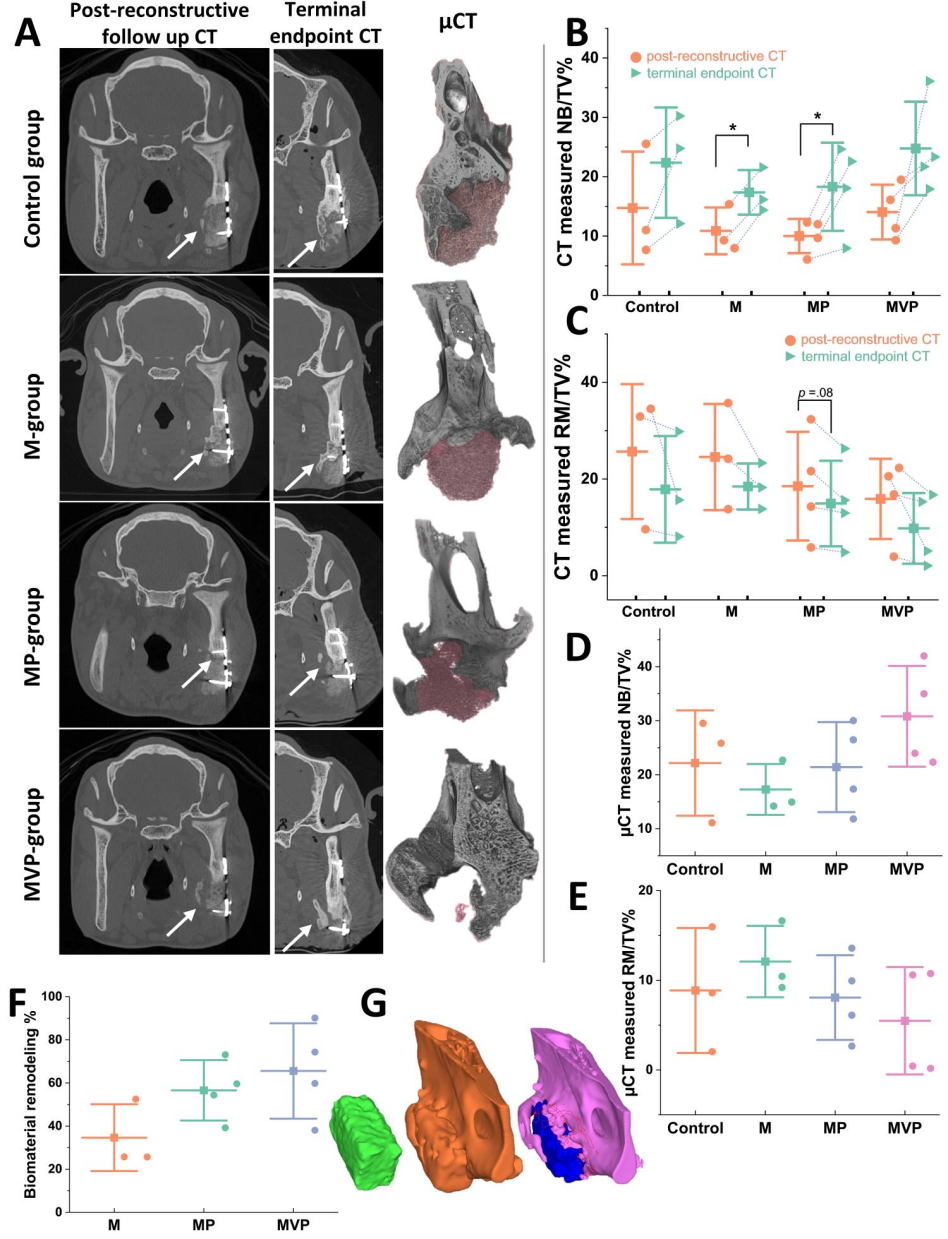
Post-reconstructive radiographic analyses results. Representations of the computed tomography (CT) and micro-CT (µCT) analyses (A). Comparing the CT images from post-reconstructive follow-up scans (left column in A) to the terminal-endpoint CTs (middle column in A) showed progressive bone defect healing and biomaterial degradation in all groups. Most of the detected new bone formation progressed from the edges and lingual aspect of the defect (white arrows). The 3-dimensional (3D) models from the µCT scans were coronally cut at the same level of the shown CT images to depict the residual biomaterial (shaded red) and the formed new bone at a higher resolution (right column in A). The least residual biomaterial was evidently seen in the MVP group. The quantification for the CT-measured newly formed bone volume (NB/TV%) revealed a trend of increasing new bone volumes between the 2 CT time points within all groups with a corresponding decrease in the residual biomaterial volumes (RM/TV%) (B, C). The µCT analysis revealed an increased mean new bone formation (NB/TV%) and the least residual biomaterial volumes (RM/TV%) in the MVP group (D, E). The 3D-constructed models from pre-, post-reconstructive, and terminal CTs were analyzed for assessing biomaterials remodeling in relation to the prefabrication technique (F, G). Representative 3D models (G) depict bone block from tissue-engineered bone (TEB) flap before transplantation (green left model), TEB reconstructed mandibular defect (gold middle model), and newly formed bone (pink) and residual biomaterial (blue) at the terminal state model (the model to the right). The 3D model comparison revealed a higher mean remodeling percentage in MP and MVP groups compared to the M group (F). The boxplots show mean (–■–), SD (whiskers), and averaged observation values (•), *P < 0.05. *n*_Control_ = 3. *n*_M_ = 3. *n*_MP_ = 4. *n*_MVP_ = 4. M, intramuscular pouch; MP, a pericranial nonvascularized graft with the muscular pouch; MVP, pericranial vascularized flap with the muscular pouch.

Post-reconstructive follow-up and terminal-endpoint CTs revealed active new bone formation and biomaterial degradation in all groups (Fig. 3A). The newly formed bone volume (NB/TV%) increased between the 2 time points within all groups. MVP group was associated with the numerically highest mean NB/TV% of 24.75% (Fig. 3B). In parallel, the residual biomaterial volume (RM/TV%) decreased in all groups, with the mean RM/TV% in the MVP group being 9.80%, which was the lowest among groups (Fig. 3C). The comparisons of the CT-reconstructed 3D models reflected the remodeling of the biomaterials within the tested IVBs, with the lowest remodeling in the M group and highest in the MVP group (Fig. 3F, G).

#### µCT analysis

The µCT analysis findings for the endpoint mandibular samples supported the CT analysis results. The largest mean differences were seen between MVP and M groups, as more new bone formation and less biomaterial volume were evident in the MVP group. However, the differences were not statistically significant due to the variable individual response (Fig. 3D, E).

#### Histological analysis

**Figure 4.**
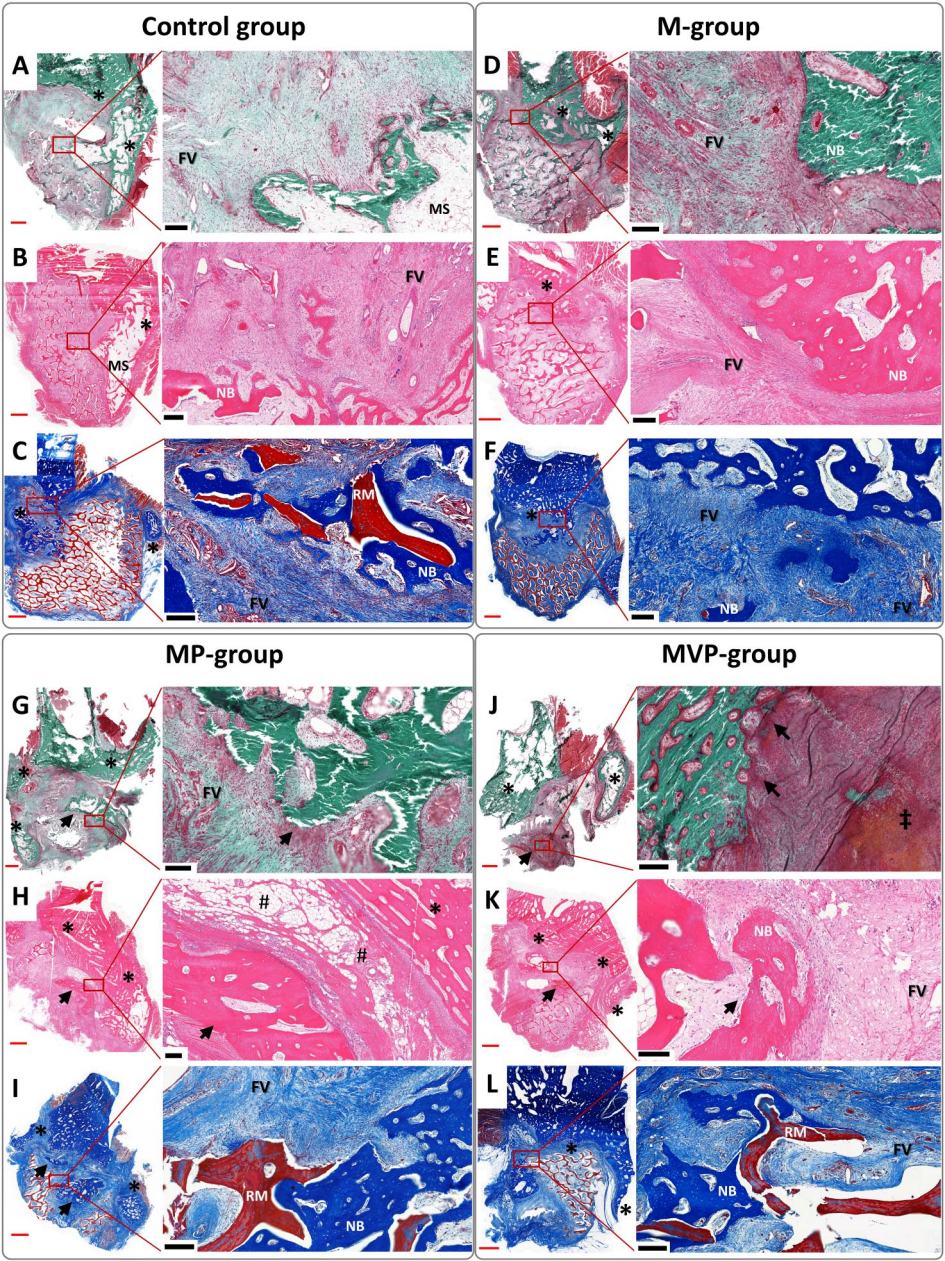
Photomicrographs represent the histological findings of the terminal-endpoint samples. The undecalcified sections were stained by Masson Goldner trichrome (A, D, G, J), and the decalcified sections were stained by hematoxylin and eosin (B, E, H, K) and Masson’s trichrome (C, F, I, L). Most of the detected new bone was seen mainly in relation to the defect edges/periosteum (*), especially lingually, with the related marrow spaces (MSs). However, bone islands (black arrows) were frequently seen in MP and MVP groups (G–L). The ingrowing intramembranous new bone (NB) enveloped areas of the residual biomaterial (RM), which was generally infiltrated with a fibrovascular stroma (FV). Some areas of mixed endochondral and intramembranous ossification were seen (‡). Perivascular fatty infiltration (#) was a common finding in the muscular components of the prefabricated tissue-engineered bone (TEB) flaps. The red scale bars in whole-slide images = 2,000 µm; black scale bars in higher magnification (of red boxes) = 200 µm. M, intramuscular pouch; MP, a pericranial nonvascularized graft with the muscular pouch; MVP, pericranial vascularized flap with the muscular pouch.

**Figure 5.**
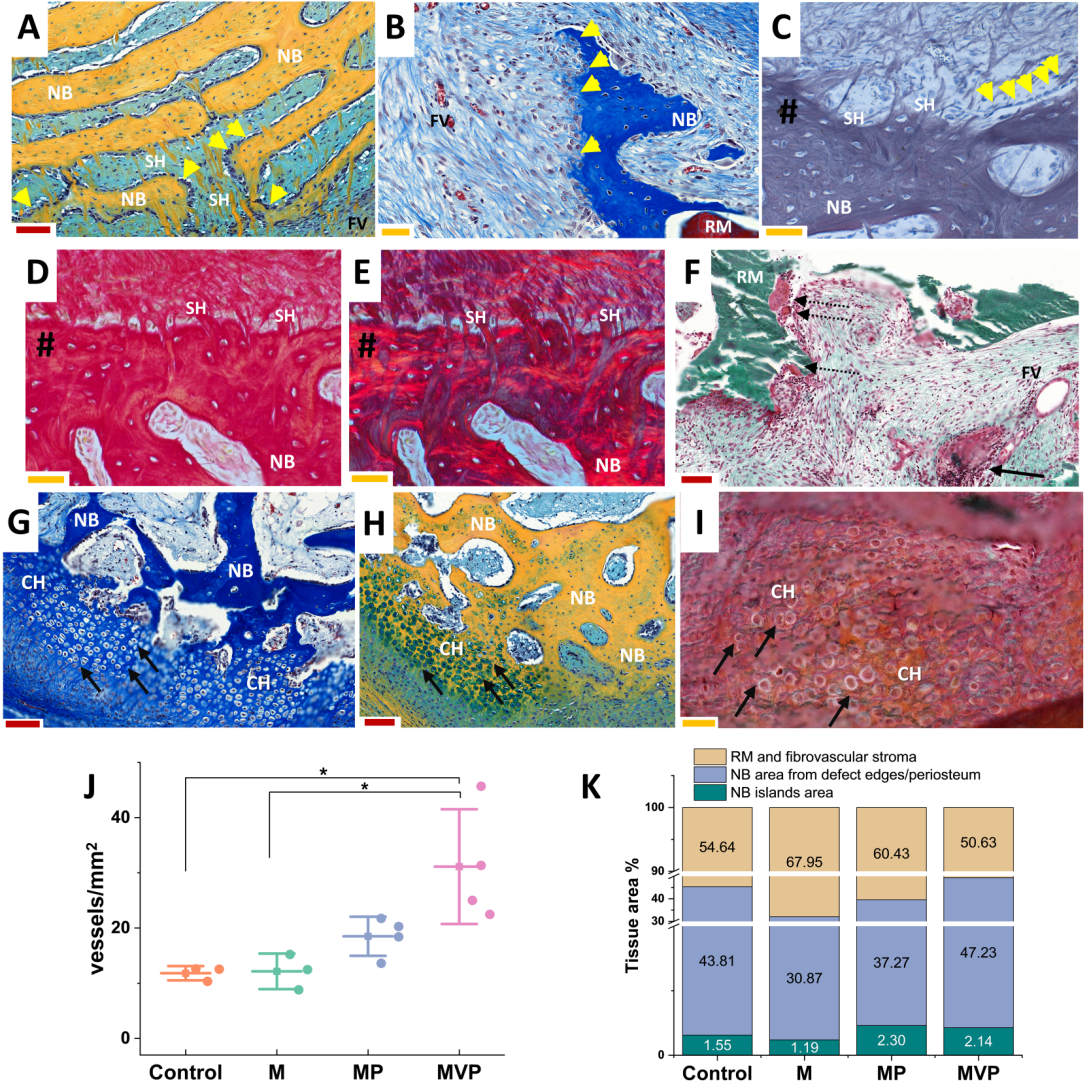
Detailed histological findings and measurements. Photomicrographs (A–E) illustrate the characteristic finding of the radiating, speckled, cellular, perforating collagen fibers (SH) related to the newly formed bone (NB). The perforating fibers (SH) showed similarities with Sharpey’s fibers, as shown in decalcified sections stained with reticulin (C) and picrosirius red under brightfield (D) and polarized light (E) (# indicates bone surface). The newly formed bone showed related osteoblastic cells (yellow arrowheads, A–C). The biomaterial degradation foci (F) showed groups of macrophages and multinucleated giant cells (dashed arrows) with remnants of biomaterial (RM), and collections of lymphocytes and plasma cells (black arrow in F) were not infrequently seen close to a nearby vascular channel within the fibrovascular stroma (FV). Areas of endochondral-like ossification were seen (black arrows in G–I), where the newly formed bone was observed to replace areas of hypertrophied nested chondrocyte-like cells (CH). The represented sections were stained by Movat’s pentachrome (A, H), Masson’s trichrome (B, G), reticulin (C), picrosirius red (D, E), and Masson Goldner trichrome for undecalcified sections (F, I). An increased density of blood vessels (vessels/mm^2^) was observed in MVP group samples compared to the control and M groups (J). The mean tissue area of the newly formed bone with its related marrow spaces was the least in the M group, while it was the largest in the MVP group (K). Larger areas of the newly formed bone islands were seen in both MP and MVP groups (K). The red scale bars (A, F–H) = 100 µm, and yellow scale bars (B–E, I) = 50 µm. The boxplot (J) shows mean (–■–), SD (whiskers), and averaged observation values (•), *P < 0.05. The stacked column chart (K) shows the average measured areas in histological sections. *n*_Control_ = 3. *n*_M_ = 3. *n*_MP_ = 4. *n*_MVP_ = 4. M, intramuscular pouch; MP, a pericranial nonvascularized graft with the muscular pouch; MVP, pericranial vascularized flap with the muscular pouch.

The individual responses within groups were heterogenous, but the new bone formation followed consistent patterns. Most of the bone formation arose from the bony edges of the defect and surrounding periosteum, especially the lingual periosteum (Fig. 4). Nevertheless, new bone islands were frequently found within the biomaterials with no connection to the native periosteum at the recipient site. New ingrowing intramembranous bone infiltrated and enveloped the biomaterial (Fig. 4C, I, L). The bone islands were more evident in both MP and MVP groups (Fig. 4G–L). Areas of mixed endochondral and intramembranous ossification were occasionally seen (Fig. 5G–I).

The M group showed the least mean new bone formation, and the MVP group showed the most (Fig. 5K). The newly formed bone with marrow spaces occupied on average 32% of the implant area (32.05% ± 10.89%) in the M group, 45% (45.36% ± 17.81%) in the control group, and 49% (49.37% ± 14.65%) in the MVP group. Segregating the newly formed bone into bone islands and bone from the defect edges/periosteum revealed mild enlargement of bone island areas in the MP and MVP groups (Fig. 5K).

The increased new bone formation in the control and MVP groups was associated with intensified degradation of the biomaterial, especially in the MVP group (Fig. 5K). New woven bone, especially originating from the defect edges, appeared more evident where biomaterial degradation was pronounced, frequently forming conspicuous lamellae and growing along transversally oriented fibers (Fig. 5A). The transversal fibers appeared to penetrate the newly formed bone, radiating toward the degrading biomaterial and its fibrovascular stroma, and showed Sharpey’s fiber–type picrosirius red and reticulin staining characteristics (Fig. 5A–E). The fibrovascular stroma filled the biomaterial spaces and was significantly more vascularized in the MVP group (Fig. 5J), whereas it was more fibrotic in the M group (Fig. 4F).

## Discussion

The periosteum plays a crucial role in physiologic bone remodeling and repair; its osteogenic potential was described 3 centuries ago (Hutmacher and Sittinger 2003; Soldado et al. 2012). Elucidating the factors that influence the periosteal inherent regenerative capacity is mandatory for a predictable application in reconstructive approaches. The pericranium is a clinically relevant source due to the feasibility for harvesting larger periosteal tissues (Battaglia et al. 2020). In this study, the ectopic employment of periosteal grafts and flaps in muscle IVBs was assessed. The periosteum showed a predictable provascularization and pro-osteogenic potential. The vascularized periosteal flaps had greater provascularization effects compared to transplanted nonvascularized grafts. Biomaterial remodeling was enhanced in association with vascularized periosteal flaps. The osteogenic potential of periosteum, however, was not critically affected by the maintenance of its own vascular supply but rather depended on its interaction with a mechanically stimulated local bony microenvironment after transplantation into mandibular defects.

Previous studies have shown variable dependence of the periosteal osteogenic capacity on contact with viable osseous tissues (Burstein and Canalis 1985; Canalis and Burstein 1985; Ersoy et al. 2015). Based on dog model studies, Canalis and Burstein (1985) suggested that the osteogenic capacity of the periosteum depends on both the maintenance of its blood supply and the interaction with a viable bone tissue. They later reported that, despite the lack of significant bone-periosteal contact, the costal periosteum showed osteogenesis when transferred onto soft tissues (Burstein and Canalis 1985). Radial periosteum was reported to be osteogenic when transplanted into canine omental or subcutaneous areas (Bigham-Sadegh et al. 2013). In our study, the pericranial grafts and vascularized flaps with embedded BBs were not capable of inducing ectopic bone formation in a muscle pouch during flap prefabrication despite achieving robust vascularization (Fig. 2). Nevertheless, after transplantation for reconstructing mandibular defects, both the periosteal graft and periosteal flap wrapped BBs induced more bone islands as compared to other groups (Fig. 4G–L). These findings suggest that the osteogenic potential of periosteal tissues is retained through the prefabrication phase and is dependent on the bone microenvironment and mechanical stimulation achieved after transplantation.

The duration for the prefabrication phase in this study was based on previous reports, which suggested an optimal duration of approximately 8 wk (Runyan et al. 2014; Kasper et al. 2017; Naujokat et al. 2020). Obviously, previous study designs were variable, but using the same duration range would allow for comparison of results. In our study, we opted to use the brachiocephalic muscle IVB due to its anatomical proximity to the pericranium, which facilitated the application of vascularized pericranial flaps. Moreover, it allowed the later harvesting of prefabricated TEB flaps as pedicled flaps for mandibular reconstruction. This design could be promising for clinical translation in selected patients for augmenting the bony component of, for example, the sternocleidomastoid flap (Wei et al. 2013; Chen and Chang 2015).

After the prefabrication phase, CTA has shown no differences in the detected BVs around the prevascularized BBs (Fig. 1B, D). However, a significantly increased BV density was histologically detected in MVP group samples (Fig. 2D–I), which could be due to the small size of BVs, which was below the detection range of CTA. The muscular pouches (M group) were associated with less vascularization, reduced new bone formation, and biomaterial remodeling. The relative increase of stromal fibrosis in the M group (Fig. 4F) supports the positive role of periosteal grafts/flaps in providing a simultaneous guided bone regeneration concept as previously reported (Dimitriou et al. 2012; Huang, Kobayashi, et al. 2016).

Biomaterial degradation is crucial to allow ingrowth of new bone and vascular tissues. Marked amounts of residual biomaterial, regardless of its biocompatibility, can later lead to complications (Heliotis et al. 2006; Sheikh et al. 2015). Wu et al. (2017) showed a higher degradation rate in β-tricalcium phosphate scaffolds with increased vascularization by a saphenous arteriovenous loop as compared to vascular bundle in beagle dogs. Correspondingly, we observed an enhanced biomaterial degradation in the MVP group (Fig. 3A, C, E, F), which also showed an increased vascularization (Figs. 2D–I, 5J). However, a considerable biomaterial degradation was found in the least-vascularized control group, which could suggest that the mechanical stresses at the recipient site play an additional principal role in biomaterial degradation.

Aaron and Skerry (1994) have suggested the role of Sharpey’s fibers in trabecular generation in developmental and regenerative bone. Our model extends these findings to bone ingrowth into implanted biomaterials. In harmony with previous reports (Aaron and Skerry 1994; Aaron 2012), perforating Sharpey’s fibers bridged the excised bony surfaces, the periosteum/endosteum, and the biomaterials, as well as exhibited a scaffolding effect for regenerating trabecular intramembranous ossification (Fig. 5A–E).

The limitations of this study include a priori overestimated effect size for the employed IVBs; the observed effects were short of statistical significance due to the variability of individual responses. In addition, the CSD design partially preserved the recipient site periosteum, which showed a regenerative capacity as manifested in the control group. Nevertheless, these findings highlight the significant impact of the recipient site periosteum and the importance of its preserving and/or in situ prefabrication. The latter is the fundamental principle of the 2-staged Masqualet induced membrane technique (Masquelet 2017). Our observed osteogenic potential of pericranial transplants at the recipient site proposes their direct application in a single-stage reconstructive approaches that would be clinically interesting and deserving future investigation.

## Author Contributions

A.G. Abu-Shahba, contributed to conception, design, data acquisition, analysis, and interpretation, drafted and critically revised the manuscript; T. Wilkman, contributed to conception, design, data acquisition, and interpretation, critically revised the manuscript; R. Kornilov, contributed to data acquisition, critically revised the manuscript; M. Adam, contributed to design and data acquisition, critically revised the manuscript; K.M. Salla, contributed to design and data acquisition, drafted and critically revised the manuscript; J. Lindén, R. Björkstrand, contributed to data acquisition, analysis, and interpretation, drafted and critically revised the manuscript; A.K. Lappalainen, contributed to data acquisition and analysis, critically revised the manuscript; R. Seppänen-Kaijansinkko, contributed to conception and design, critically revised the manuscript; B. Mannerström, contributed to conception, design, data acquisition, and interpretation, drafted and critically revised the manuscript. All authors gave final approval and agree to be accountable for all aspects of the work.

## Supplementary Material

Supplementary material

Supplementary material
