# Supplementary material for: Periosteal Flaps Enhance Prefabricated Engineered Bone Reparative Potential
Source: J Dent Res. 2021 Sep 11;101(2):166–76. doi: 10.1177/00220345211037247 (PMC8808084; doi:10.1177/00220345211037247)
Supplement: Supplementary material [file sj-pdf-1-jdr-10.1177_00220345211037247.pdf]

Supplementary Appendix

Periosteal flaps enhance prefabricated engineered bone reparative potential

Ahmed Abu-Shahba [1,2], Tommy Wilkman [3], Roman Kornilov [1], Magdy Adam [4], Kati Maria Salla [4], Jere Lindén [5,6], Anu Lappalainen[4], Roy Björkstrand [7], Riitta Seppänen-Kaijansinkko [1,3], Bettina Mannerström [1]

[1] Department of Oral and Maxillofacial Diseases, University of Helsinki and Helsinki University Hospital, Helsinki, Finland

[2] Department of Oral and Maxillofacial Surgery, Faculty of Dentistry, Tanta University, Tanta, Egypt

[3] Department of Oral and Maxillofacial Surgery, Helsinki University Hospital, Helsinki, Finland

[4] Department of Equine and Small Animal Medicine, Faculty of Veterinary Medicine, University of Helsinki, Helsinki, Finland

[5] Department of Veterinary Biosciences, Faculty of Veterinary Medicine, University of Helsinki, Helsinki, Finland

[6] Finnish Centre for Laboratory Animal Pathology (FCLAP), HiLIFE, University of Helsinki, Helsinki, Finland

[7] Department of Mechanical Engineering, Aalto University, Finland

1 General anesthesia (GA) and perioperative management protocol

1.1 First stage surgery (prefabrication in IVBs phase)

Sheep were premedicated with intramuscular xylazine (0.2 mg/kg) and ketamine (1 mg/kg) or acepromazine (0.03 mg/kg), midazolam (0.3 mg/kg) and butorphanol (0.2 mg/kg). GA was induced with intravenous ketamine (2 mg/kg) and propofol (2 mg/kg) followed by endotracheal intubation. During the surgical procedure, sheep were kept on left lateral recumbency and anesthesia was maintained with sevoflurane in oxygen (ETsevo 2.3%). Intermittent positive pressure ventilation was provided as needed to target of end-tidal CO_2 ($ETCO_2$) 35 - 45 mmHg. During the anesthesia, standard clinical and instrumental monitoring were carried out. Prior to incision, local anesthesia with lidocaine cum adrenaline (maximum 4 mg/kg of lidocaine) was applied. During the surgery, rescue analgesia (fentanyl 3 µg/kg IV) was given if needed. Perioperative fluid therapy with Lactated Ringer (5-10 ml/kg/h) was infused throughout the anesthesia. Prophylactic antibiotic therapy (ampicillin 22mg/kg IV) was administered 30 minutes prior to incision and every 90-minute interval until the end of surgery.

After the surgery sheep were returned to the group pen, when they recovered from anesthesia and had normal locomotor activity. Post-operative analgesia consisted of repeated intramuscular buprenorphine (0.006-0.008 mg/kg) and fentanyl patch (2 µg/kg) applied to the antebrachium according to the experimental animal license.

1.2 Pre-reconstructive CT scanning (1st CT) and reconstructive surgery (2nd surgery)

After the premedication with intramuscular butorphanol (0.3 mg/kg) and midazolam (0.3 mg/kg), anesthesia was induced with intravenous ketamine (2 mg/kg) and propofol (2 mg/kg) followed by endotracheal intubation. During the CT scanning and mandibular surgery, GA was maintained with the same protocol as previously detailed in 1st surgery. The intraoperative analgesia, perioperative fluid therapy, prophylactic antibiotic, and post-operative analgesia were performed in the 1st surgery protocol.

1.3 Follow up post-reconstructive CT scanning (2nd CT)

Sheep were premedicated with intramuscular medetomidine (40 µg/kg) and ketamine (1 mg/kg). Anesthesia was induced and maintained with intermittent intravenous boluses of ketamine (1-2 mg/kg) and propofol (1-2 mg/kg) as needed. Oxygen was supplied with facemask. Intravenous fluid therapy with Lactated Ringer's solution (10 ml/kg/h) was administered throughout the procedure. Sheep were returned to the group pen, when they recovered from anesthesia and had normal locomotor activity.

1.4 Terminal sedation

Sheep were sedated with intravenous xylazine (0.5mg/kg) with or without vatinoxan (750 µg/kg). When appropriate sedation was achieved, approximately at 10 minutes later, sheep were euthanized with pentobarbital (100 mg/kg) IV.

2 The surgical procedures

2.1 First stage surgery (prefabrication in IVBs phase)

All the surgical procedures were performed in the operating theatres of the Equine and Small Animal Medicine, Veterinary Teaching Hospital, University of Helsinki. Food was withheld for at least 12 hours prior to any intervention under GA, with water accessible *ad libitum*. TW performed the surgeries together with AA. The GA was performed by veterinary anesthesiologists (KS and MA). The details for the premedication, GA, perioperative fluids, prophylactic antibiotic, and analgesia protocol is provided in the supplementary appendix; section 1.

Under GA, with the sheep put to a left lateral recumbency position, the fleece over the right aspect of the neck and forehead was trimmed and the skin of the surgical field was carefully disinfected, prepped, and draped in a sterile fashion. Prior to incision, the tissues were infiltrated with local anesthetic (LA) lidocaine cum adrenaline (maximum 4 mg/kg of lidocaine). A lazy S incision was carried out on the dorsal right aspect of the neck, a rostral extension of the incision was performed over the forehead when the exposure of the pericranium was needed in MP and MVP groups (Appendix Fig. 1D). Sharp and blunt dissection with meticulous hemostasis was performed through subcutaneous tissues followed by the creation of the brachiocephalic muscular pouch (Appendix Fig. 1A, B) at its rostral part ventral to the splenius muscle. The bone blocks (BBs) were implanted into the muscular pouches in M-group sheep after soaking in venous blood (Appendix Fig. 1C). In MP-group sheep, a nonvascularized pericranial graft was harvested for wrapping the blood-soaked BB with the cambium layer facing inwards (Appendix Fig. 1D-I). In MVP-group, an axial pericranial vascularized flap based on branches of the occipital, posterior auricular, and posterior meningeal arteries was raised (Appendix Fig. 1J). The blood-soaked BB was wrapped with the vascularized pericranial flap facing its cambium layer. In both MP- and MVP-group, the pericranium-wrapped BBs were implanted in a similar muscular pouch as done in M-group (Appendix Fig. 1I, K). After infiltrating tissues with 5 ml long-acting LA (Ropivacain, 10 mg/ml, Fresenius Kabi AB), the muscular pouch and subcutaneous tissues were closed by resorbable Vicryl 3-0 suture (Ethicon), and the skin was closed by 2-0 Ethilon suture (Appendix Fig. 1L) to be removed 10 days postoperatively.

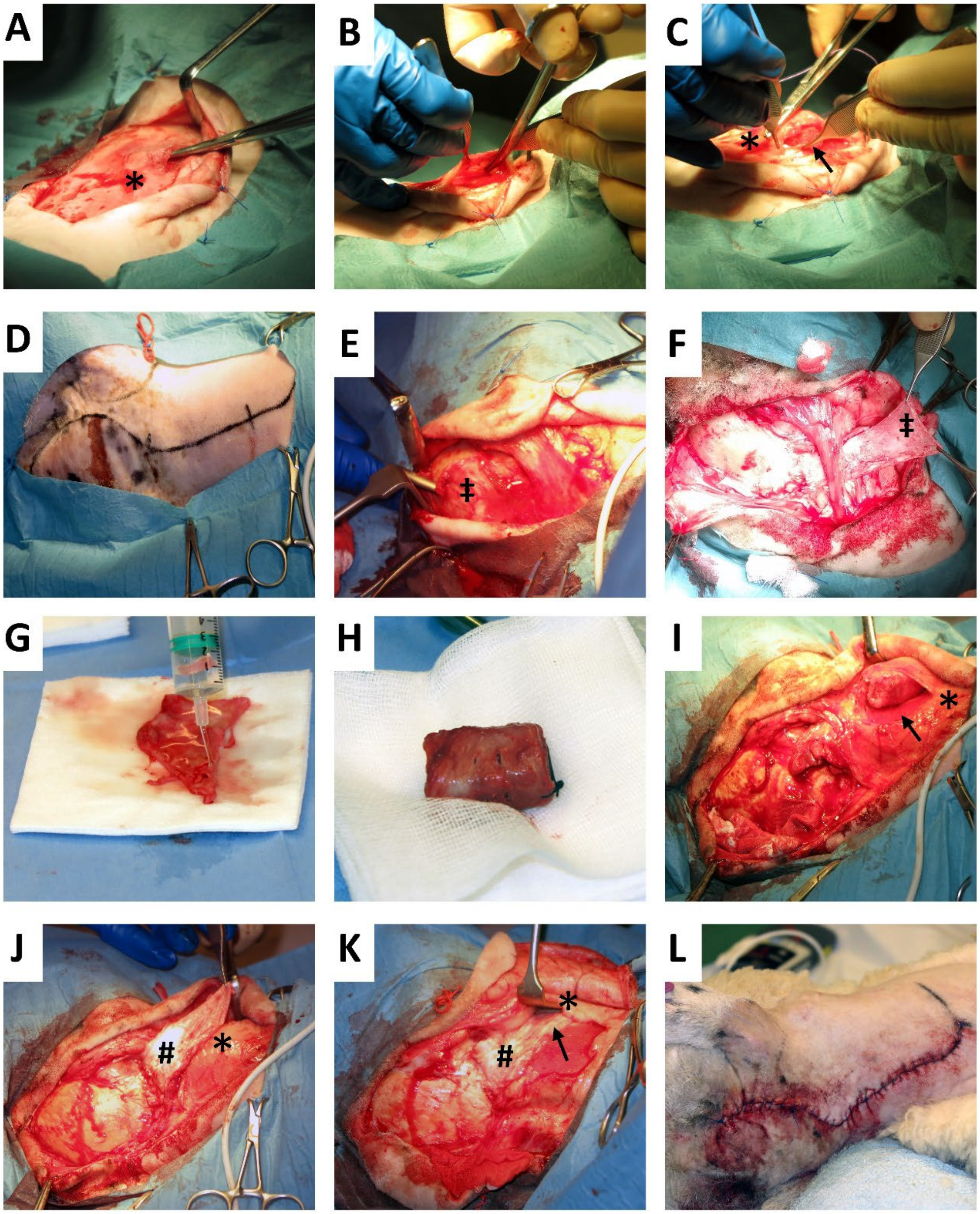

Appendix Fig. 1: The first surgery for implanting the bone blocks (BBs) into the tested IVBs, i.e., muscle pouch (M) (A-C), pericranial graft with muscle pouch (MP) (D-I), or pericranial flap with muscle pouch (MVP) (J-L). In all the sheep, the muscular pouch (arrow) was created in the brachiocephalic muscle (✱) (A and B). In M-group, the BBs were inserted directly into the pouch (C). In MP-group, a periosteal/pericranial graft (‡) was elevated by a periosteal elevator (E), harvested (F and G) to wrap the BBs (H) before implanting in the muscular pouches (arrow) (I). In MVP-group, periosteal/pericranial vascularized flaps (#) were elevated to wrap the BBs before implanting in the muscular pouches (J and K). The surgical wound was closed in layers (L).

2.2 Second stage surgery (reconstructive phase)

After the pre-reconstructive CT scans, the sheep were immediately moved to the operating room where they were prepared for the second surgery under GA by positioning, prepping, and draping in a sterile fashion, as previously described, exposing the right dorsolateral aspect of the neck and the right submandibular region. Similar to the first surgery, the same protocol for LA infiltration was applied. A lazy S incision was performed on the right lateral aspect of the neck with a submandibular extension towards the right mandibular angle region. Sharp and blunt dissection with careful hemostasis was performed to expose superficial neck muscles. The prefabricated TEB flap was raised by sharp dissection through the brachiocephalic muscle to include the prevascularized BB with a surrounding muscle tissue as a myoosseous flap (Appendix Fig. 2). The flap dissection involved the preservation of the pedicle which comprises occipital artery branches to the muscle segment (Appendix Fig. 2A, B). These branches showed a consistent close relation to the accessory nerve. The right mandibular angle was exposed through the sharp dissection of the pterygomasseteric sling. A CSD corresponding to the intraoperative dimensions of the BB in the prefabricated TEB flap was created using a bone saw (Stryker or DePuy Synthes) under saline irrigation. The defects were 29 (±2) × 18 (±1) mm in average. The transplanted flap was used for CSD reconstruction after careful exposure of the BB surfaces which faced the CSD edges (Appendix Fig. 2C-E). The internal fixation was accomplished by a miniplate and screws (Appendix Fig. 2E). In three randomly assigned sheep (one sheep/IVB), the prevascularized BBs were harvested for histological analysis and the CSDs were reconstructed using fresh blood-soaked BB, and these constituted the control group. The surgical wound was closed in layers with resorbable 3-0 Vicryl and PDS-II (Ethicon).

2.3 The recovery and postoperative course

All sheep recovered from the surgical procedures under GA without complications. Generally, sheep were able to return to normal activity, diet, and rumination after the recovery. However, one week after the first surgery, one sheep from M-group showed dyspnea and decreased activity. The sheep was euthanized based on veterinarian decision; the necropsy revealed a previously undiagnosed ventricular septal defect.

During the postoperative course, the expected postoperative edema of the surgical wound was seen with no signs of distress or pain under the implemented analgesic protocol. Generally, surgical wound healing was uneventful with no signs of infection throughout the follow up period. However, one sheep (M-group) developed a seroma after the first surgery, which resolved after aseptic aspiration. After the second surgery, another sheep (M-group) had a partial acute wound disruption in the neck due to a thrust by another sheep. The wound was managed by local debridement and allowed to heal secondarily under antibiotic coverage (ampicillin IV, A-Pen; 1g bid, Orion Pharma). A

sheep from MVP-group developed a late local inflammation in the retromandibular region 6 weeks after the second surgery. The inflammation resolved under antibiotics (ampicillin IV, A-pen; 1g bid) for four days.

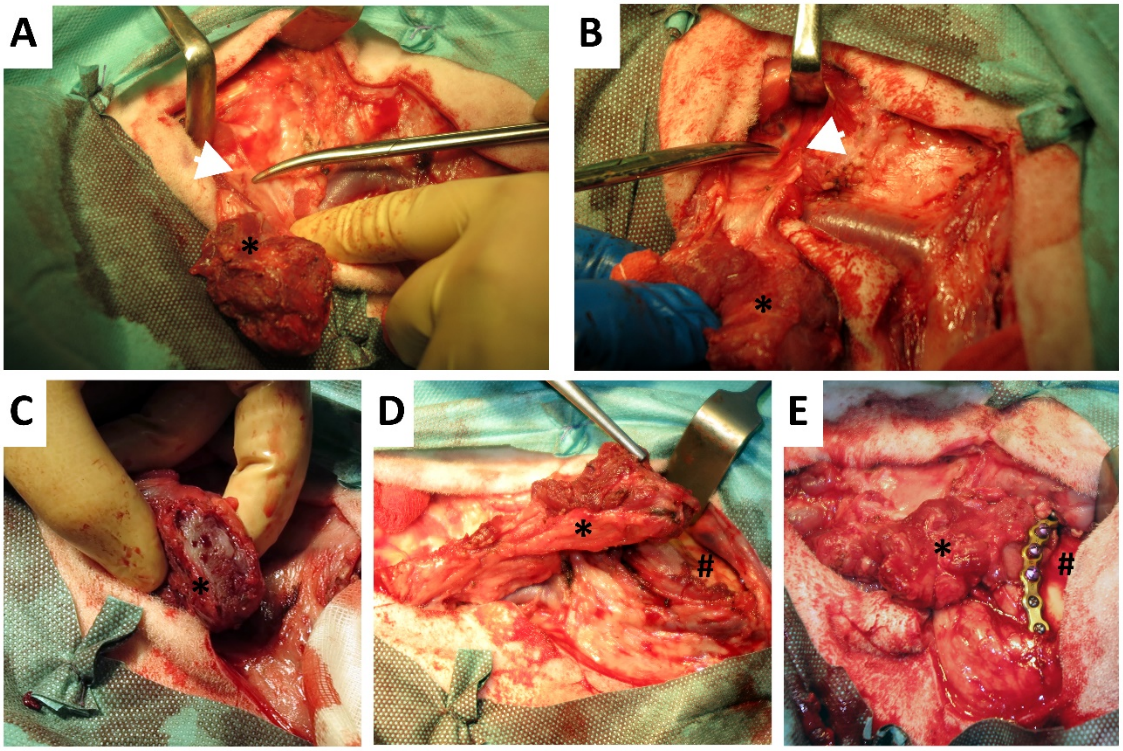

Appendix Fig. 2: The second (reconstructive) surgical phase. The instrument and white arrow heads show the vascular pedicle during raising the prefabricated TEB flap (✱) (A and B). Evident vascularization through the biomaterial pores was seen intraoperatively (C). The prefabricated TEB flap (✱) was transplanted for reconstruction of mandibular angle (#) bone defect (D and E).

3 The CT and µCT scans

Under GA, each sheep underwent CT scan of the head and neck, first without and then with IV contrast material (CT angiography, CTA). The scans were performed by a LightSpeed VCT 64 slice CT Scanner (GE Medical Systems, USA). The contrast material (Omnipaque 300mg I/ml, Oy GE Healthcare Bio-Sciences Ab) was injected IV via a cannula in the cephalic vein, at 2 ml/kg with a rate of 3 ml/s using a power injector. The scanning parameters for pre-reconstructive CT involved the settings for voltage at 120 KV; a maximum of 698 mAs; 0.625 mm slice thickness, rotation time of 0.5s; and a total collimation width of 40 mm. For decreasing plates and screws artefacts, the parameters for the post-reconstructive and terminal-point CT were set at a voltage peak of 140 KV; a maximum of 609 mAs; 0.625 mm slice thickness, rotation time of 0.7s; and a total collimation width of 40 mm.

For the µCT scanning, a GE phoenix nanotom s system (General Electric Sensing and Inspection Technologies/Phoenix X-ray, Germany) was used. The samples were imaged at 50.0 µm voxel size, with X-ray generator settings at 80 kV and 150 µA, using a 1 mm Al filter. A total of 1200 projection images were recorded over a 360-degree rotation of the sample with 3 x 500 ms exposure time for each projection. The 3D volume data was reconstructed from these data sets using Bruker NRecon version 1.6.10.2 (Bruker, Belgium).

4 Methodology for CT and µCT analysis

For the CT analyses, each DICOM dataset was loaded into CTAnalyser (CTAn) software 1.18.8.0 (Bruker, Belgium). For each dataset (sheep/timepoint), two volumes of interest (VOI) were manually registered, one for the residual biomaterial (RM), defined by its geometry and pattern, and the second VOI was for the newly formed bone (NB) continuous with the edges of the defect (Appendix Fig. 3A, B). On the binary selection preview, the histograms from the dataset helped to perform the greyscale thresholding. The lower limit was set within the valley of the bimodal histogram which achieved the least noise, the upper limit was adjusted at a level less than the maximum to exclude the plates and screws from the analysis. Subsequently, the 3D analysis was performed on the custom processing preview after global thresholding with the preset greyscale levels and despeckling to remove white speckles less than 100 voxels in 3D space and applied to image.

In a parallel setting, the change in the volume of the BB was evaluated by comparing the 3D reconstructed models from datasets of each timepoint to estimate the resorbed volume at the terminal endpoint as compared to the initial pre-reconstruction volume. The detailed protocol for the 3D-model reconstruction and volume comparison is provided in the next section (section 5).

For the µCT analysis, the datasets were first loaded into DataViewer 1.5.4.0 (Bruker, Belgium) for reorientation, all the datasets were sagittally reoriented for consistency and saved as new datasets. Further processing was performed on CTAn software, the new datasets were loaded and two VOIs were registered for each dataset. The RM-VOI included the residual biomaterial based on its characteristic pattern, the NB-VOI included the newly formed bone within the corresponding dimensions of the reconstructed defect. Those dimensions were recorded based on the excised bone pieces and verified in the mid-sagittal plane of the reconstructed 3D model in CTvox 3.3.0 (Bruker, Belgium) (Appendix Fig. 3C, D). Since the µCT scanned samples did not have the plates and screws, automatic thresholding was feasible. The 3D analysis of each VOI was performed in the custom processing preview of CTAn after automatic thresholding (Otsu's method) and subsequent despeckling to remove white speckles less than 1000 voxels in 3D space and applied to image.

5 Methodology for CT-3D models comparison

The sheep CT data was 3D reconstructed using open source 3DSlicer software (www.slicer.org). The volumes reconstructed for the analysis were: pre-reconstruction (Pre-vol), post-reconstruction (Post-vol), and terminal volume (End-vol). The 3D models were further cleaned from noise and extra objects using 3DataExpert software (DeskArtes Oy, Finland).

Volume of Interest (VOI) is defined as the 3D volume reconstructed by 3DSlicer algorithm using the threshold value of 300, lower bone density value, and smoothing factor 0.5. The volumetric reconstruction is an interpretation of grey-value voxels defined by CT-image stacks and defines the geometry of *bone dense* parts of object. Tissues with lower threshold than 300 were considered as empty space. Two VOIs were used in this analysis; reconstruction volume as-is and reconstruction volume solidified, small holes filled by manipulating the faceted geometry in 3DataExpert. Two geometries were used to validate the method for porous VOI that was seen in some of cases.

Since different CT based geometries naturally were in different coordinates, the reconstructed 3D models were aligned into same spatial orientation. For reorientation, open source CloudCompare software (cloudcompare.org) was used. Post-vol reconstruction was used as placeholder for 3D-models. Both Pre-vol and End-vol were oriented using landmarks of Post-vol. Reorientation was done using Post-vol due to assumingly more accurate volumetric correspondence with Pre-vol. Post-vol and End-vol were aligned using mandible landmarks to reach best accuracy. The functions used for reorientation were three-point registration and freehand translate/rotation (Appendix Fig. 4A).

Reoriented 3D model volumes were measured using 3DataExpert (DeskArtes Oy, Finland). The volume that was reconstructed from Pre-vol CT was used to delimit a corresponding spatial region in terminal state to find the volume of bone dense parts of End-vol inside VOI. Using Boolean operator – subtracting volumes – it was possible to calculate the volume of End-vol located inside the VOI, thus, it was possible to estimate the remodeling percentage (Appendix Fig. 4B).

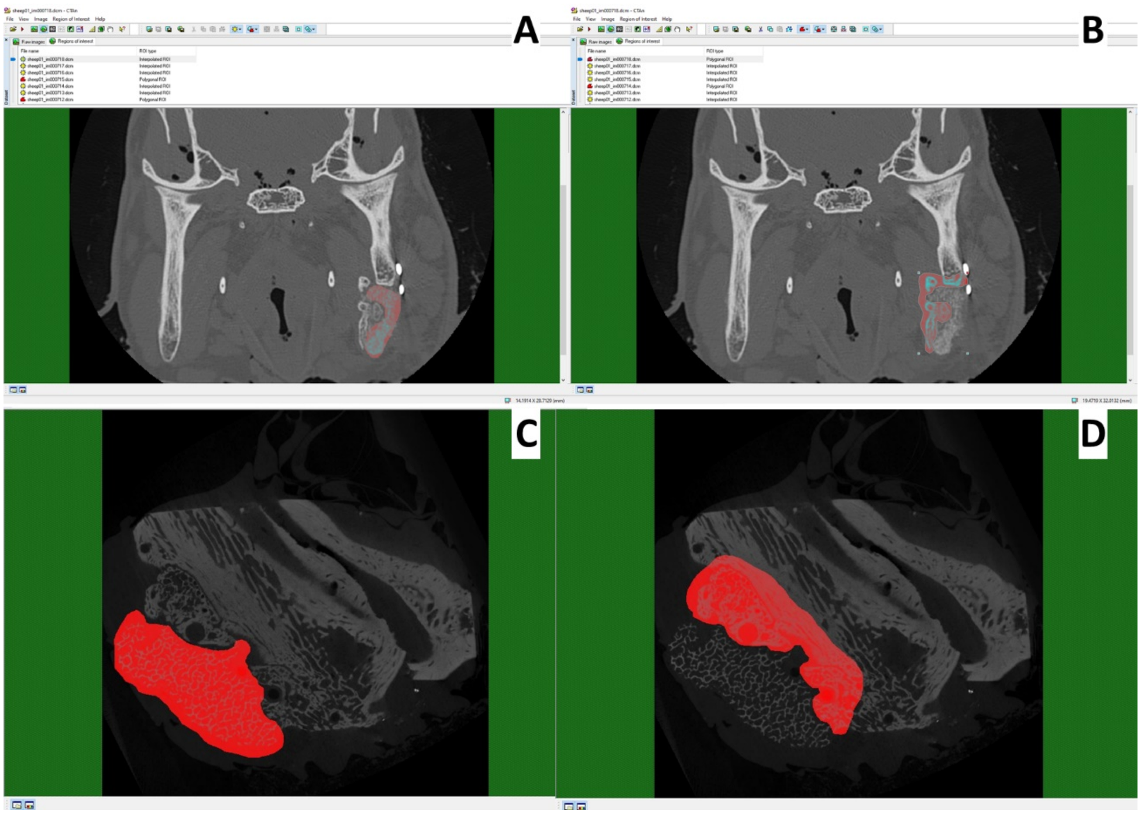

Appendix Fig. 3: Representative images for the registration of regions of interest (ROIs) for analyzing CT (A and B) and µCT (C and D) data volumes of interest (VOIs). On CTAn software, two ROIs were drawn per slice in the slices covering the reconstructed defect in each sheep. The ROIs were for both the residual biomaterial (red-shaded in A and C) and the newly formed bone (red-shaded in B and D).

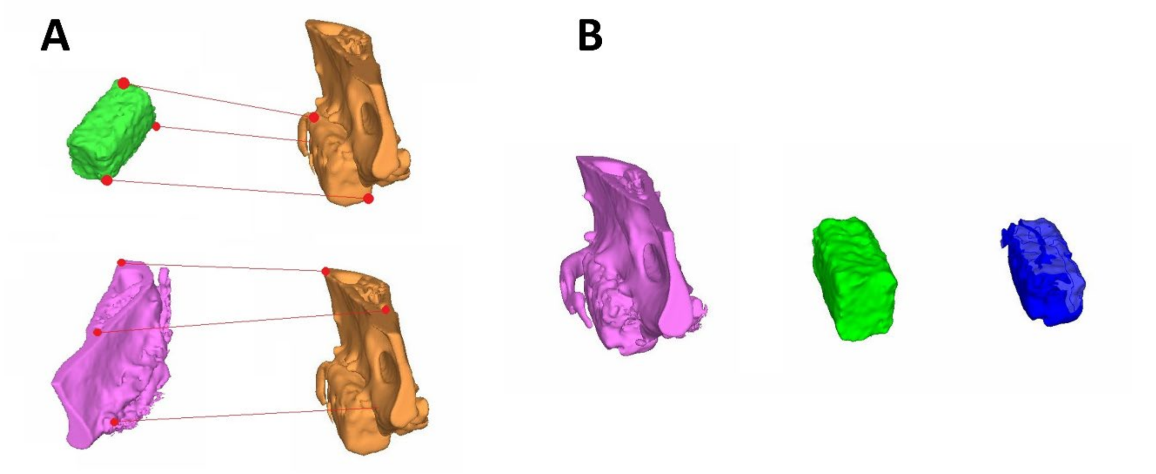

Appendix Fig. 4: Illustrations of the orientation and alignment of models for Boolean operation. The reconstructed models were aligned using Post-vol (gold) as a placeholder for both Pre-vol (green) and End-vol (pink) (A). Once the Pre-vol (green) and End-vol (pink) were aligned to share same coordinates, the End-vol was Boolean intersected with Pre-vol resulting in the volume at terminal state within original state TEB volume (blue) (B).

6 Methodology of histological preparation of samples

6.1 Immunohistochemistry on paraffin embedded sections

After heat-induced antigen retrieval (20 minutes at 99°C in 10 mM citrate buffer, pH 6), the IHC utilized the anti-von Willebrand factor (vWF) antibody (1:1000; rabbit polyclonal, Ab6994, Cambridge, UK), detected with polymer-linked secondary antibody and peroxidase (BrightVision + Poly-HRP kit, ImmunoLogic, Duiven, Netherlands) and DAB chromogen according to the manufacturer's instructions. For the quantification of the percentage of DAB-positive cells/total cells, the scanned slides were processed in the open-source digital pathology software QuPath version 0.2.3 (Bankhead et al. 2017). Additionally, the vessels were counted in CaseViewer (2.4, 3DHISTECH, Budapest, Hungary) at 10× in seven arbitrary circular fields of 1.1 mm^2 surface area/field.

6.2 Undecalcified resin embedded sections processing

The formalin-fixed samples were dehydrated in ascending alcohol series, cleared in xylene, and embedded gradually into methyl methacrylate (MMA). The 5µm-thin slices were sectioned with a hard tissue microtome (Leica, SM2500 Large Scale, Heavy duty Sectioning System) and collected on albumin-glycerin coated slides. The slides were heated at +60°C for 3-5 days after sectioning for better adherence of the sections to the slides. The sections were then stained by Masson Goldner Trichrome (MT) stain.

7 Statistical analysis reporting tables

Appendix Table 7.1: Statistical analysis for the IVB vascularization scale on preoperative CTA

Descriptive statistics for the IVB vascularization scaling on preoperative CTA

IVB condition	N Analysis	Mean	Standard Deviation	SE of Mean
M	4	2.25	0.6455	0.32275
MP	5	2	1	0.44721
MVP	5	2	0.70711	0.31623

Homogeneity of Variance test: Levene's test (Absolute deviations)

	DF	Sum of Squares	Mean Square	F Value	Prob>F
Model	2	0.21429	0.10714	0.94286	0.41885
Error	11	1.25	0.11364		

At the 0.05 level, the population variances are not significantly different.

One Way ANOVA for the IVB vascularization scaling on preoperative CTA

	DF	Sum of Squares	Mean Square	F Value	Prob>F	R-square	η²	ω^2
Model	2	0.17857	0.08929	0.13547	0.87474	0.02404	0.0240383	0
Error	11	7.25	0.65909					
Total	13	7.42857						

Appendix Table 7.2: Statistical analysis for the bone block volumes at the end of prefabrication phase

Descriptive statistics for the bone block volumes after prefabrication (RM/TV%)

IVB condition	N Analysis	Mean	Standard Deviation	SE of Mean
M	4	48.58328	14.42804	7.21402
MP	5	48.15132	11.0101	4.92386
MVP	5	50.77188	13.39697	5.99131

Homogeneity of Variance test: Levene's test (Absolute deviations)

	DF	Sum of Squares	Mean Square	F Value	Prob>F
Model	2	35.39143	17.69571	0.36093	0.70498
Error	11	539.30982	49.02817		

At the 0.05 level, the population variances are not significantly different.

One Way ANOVA for the bone block volumes at the end of the prefabrication phase

	DF	Sum of Squares	Mean Square	F Value	Prob>F	R-square	η²	ω^2
Model	2	19.37256	9.68628	0.05831	0.94365	0.01049	0.0104905	0
Error	11	1827.30874	166.11898					
Total	13	1846.68129						

Appendix Table 7.3: Statistical analysis for the IHC of vWF (positive/total cells%)

Descriptive statistics for the IHC (vWF positive cells/total cells %)

	N Analysis	Mean	Standard Deviation	SE of Mean
M	7	13.98892	4.65449	1.75923
MP	7	19.15433	4.37695	1.65433
MVP	7	20.15669	2.11938	0.80105

N in this analysis refers to the different sections/blocks from all parts of the sampled blocks at the end of prefabrication phase

Homogeneity of Variance test: Levene's test (Absolute deviations)

	DF	Sum of Squares	Mean Square	F Value	Prob>F
Model	2	24.55285	12.27642	3.37721	0.05683
Error	18	65.43141	3.63508		

At the 0.05 level, the population variances are not significantly different.

One Way ANOVA for the vWFpositive/total cells %

	DF	Sum of Squares	Mean Square	F Value	Prob>F	R-square	η²	ω^2
Model	2	153.36398	76.68199	5.07673	0.01785	0.36065	0.360647	0.2797
Error	18	271.88285	15.1046					
Total	20	425.24683						

Bonferroni means comparisons

	MeanDiff	SEM	t Value	Prob	Alpha	Sig	LCL	UCL
MP M	5.16541	2.0774	2.48647	0.06883	0.05	0	-0.31716	10.648
MVP M	6.16776	2.0774	2.96898	0.02466	0.05	1	0.6852	11.65
MVP MP	1.00235	2.0774	0.4825	1	0.05	0	-4.48021	6.4849

Appendix Table 7.4: Statistical analysis for the blood vessels density (vessels/mm²) at the end of the prefabrication phase

Descriptive statistics for the IHC (number of blood vessels/mm²)

	N Analysis	Mean	Standard Deviation	SE of Mean
M	7	16.42857	4.70309	1.7776
MP	7	19.35714	3.13202	1.18379
MVP	7	23.14286	5.71339	2.15946

N in this analysis refers to the different sections/blocks from all parts of the sampled blocks at the end of prefabrication phase

Homogeneity of Variance test: Levene's test (Absolute deviations)

	DF	Sum of Squares	Mean Square	F Value	Prob>F
Model	2	12.07191	6.03596	0.9481	0.406
Error	18	114.59475	6.36638		
At the 0.05 level, the population variances are not significantly different.					

One Way ANOVA for the blood vessels density among groups

	DF	Sum of Squares	Mean Square	F Value	Prob>F	R-square	η²	ω^2
Model	2	158.64286	79.32143	3.68529	0.04555	0.29052	0.2905167	0.2037
Error	18	387.42857	21.52381					
Total	20	546.07143						

Bonferroni means comparisons

	MeanDiff	SEM	t Value	Prob	Alpha	Sig	LCL	UCL
MP M	2.92857	2.47985	1.18095	0.75898	0.05	0	-3.61611	9.4733
MVP M	6.71429	2.47985	2.70754	0.04326	0.05	1	0.1696	13.259
MVP MP	3.78571	2.47985	1.52659	0.43273	0.05	0	-2.75897	10.33

Appendix Table 7.5: Statistical analysis for the CT measured NB/TV% in the post-reconstructive follow up

Descriptive statistics for the CT measured new bone formation NB/TV% at first follow up point (post-reconstructive)

Reconstructive gp.	N Analysis	Mean	Standard Deviation	SE of Mean
control_post	3	14.72904	9.49347	5.48106
M_post	3	10.87068	3.94528	2.27781
MP_post	4	10.00965	2.8762	1.4381
MVP_post	4	14.04763	4.6115	2.30575

Homogeneity of Variance test: Levene's test (Absolute deviations)

	DF	Sum of Squares	Mean Square	F Value	Prob>F
Model	3	47.57323	15.85774	3.63318	0.05257
Error	10	43.64703	4.3647		
At the 0.05 level, the population variances are not significantly different.					

One Way ANOVA for the CT measured NB/TV% on post-reconstructive time point

	DF	Sum of Squares	Mean Square	F Value	Prob>F	R-square	η²	ω^2
Model	3	56.98011	18.99337	0.63312	0.61032	0.15962	0.159618	0
Error	10	299.99781	29.99978					
Total	13	356.97792						

Appendix Table 7.6: Statistical analysis for the CT measured NB/TV% in the terminal post-reconstructive follow up point

Descriptive statistics for the CT measured new bone formation NB/TV% at second follow up point (terminal endpoint)

Reconstructive gp.	N Analysis	Mean	Standard Deviation	SE of Mean
NB_control_end	3	22.35915	9.2922	5.36486
NB_M_end	3	17.36185	3.74946	2.16475
NB_MP_end	4	18.30101	7.42927	3.71463
NB_MVP_end	4	24.75492	7.88538	3.94269

Homogeneity of Variance test: Levene's test (Absolute deviations)

	DF	Sum of Squares	Mean Square	F Value	Prob>F
Model	3	26.15918	8.71973	0.58751	0.63689
Error	10	148.41885	14.84188		
At the 0.05 level, the population variances are not significantly different.					

One Way ANOVA for the CT measured NB/TV% on terminal endpoint time point

	DF	Sum of Squares	Mean Square	F Value	Prob>F	R-square	η²	ω^2
Model	3	130.29833	43.43278	0.78551	0.52885	0.19071	0.1907108	0
Error	10	552.92644	55.29264					
Total	13	683.22477						

Appendix Table 7.7: Statistical analysis for the CT measured NB/TV% between two follow up points

Paired sample t test for each group between the two follow up time points regarding CT measured N

Control gp		N	Mean	SD	SEM	Median	Hedge's g	cohen's *d*
NB_control_post		3	14.72904	9.49347	5.48106	11.00493	1.1744933	1.4385
NB_control_end		3	22.35915	9.2922	5.36486	24.75822		
	Difference	3	-7.63011	5.30438	3.06248	-4.69663		
	Overall	6	18.54409	9.38371	3.83088	18.43044		
	t Statistic	DF	Prob>|t|					
	-2.49148	2	0.13033					

M-gp		N	Mean	SD	SEM	Median	Hedge's g	cohen's *d*
NB_M_post		3	10.87068	3.94528	2.27781	9.28603	15.941367	19.524
NB_M_end		3	17.36185	3.74946	2.16475	16.14293		
	Difference	3	-6.49118	0.33247	0.19195	-6.40944		
	Overall	6	14.11626	4.94875	2.02032	14.86772		
	t Statistic	DF	Prob>|t|					
	-33.81632	2	8.73E-04					

MP-gp		N	Mean	SD	SEM	Median	Hedge's g	cohen's *d*
NB_MP_post		4	10.00965	2.8762	1.4381	10.84129	1.5707607	1.8138
NB_MP_end		4	18.30101	7.42927	3.71463	20.31765		
	Difference	4	-8.29136	4.57137	2.28569	-9.47636		
	Overall	8	14.15533	6.84411	2.41976	12.1432		
	t Statistic	DF	Prob>|t|					
	-3.62751	3	0.03606					

		N	Mean	SD	SEM	Median	Hedge's g	cohen's *d*
NB_MVP_post		4	14.04763	4.6115	2.30575	13.70252	0.9678709	1.1176
NB_MVP_end		4	24.75492	7.88538	3.94269	22.50888		
	Difference	4	-10.7073	9.58061	4.7903	-7.11138		
	Overall	8	19.40127	8.27758	2.92657	18.70258		
	t Statistic	DF	Prob>|t|					
	-2.2352	3	0.11146					

Appendix Table 7.8: Statistical analysis for the CT measured RM/TV% in the first post-reconstructive follow up point

Descriptive statistics for the CT measured residual biomaterial RM/TV% at first follow up point (post-reconstructive)

Reconstructive gp.	N Analysis	Mean	Standard Deviation	SE of Mean
contol_post	3	25.67449	13.94549	8.05143
M_post	3	24.54738	10.97626	6.33715
MP_post	4	18.52147	11.23636	5.61818
MVP_post	4	15.8889	8.29575	4.14788

Homogeneity of Variance test: Levene's test (Absolute deviations)

	DF	Sum of Squares	Mean Square	F Value	Prob>F
Model	3	40.23291	13.41097	0.48603	0.69952
Error	10	275.9311	27.59311		

At the 0.05 level, the population variances are not significantly different.

One Way ANOVA for the CT measured RM/TV% on post-reconstructive time point

	DF	Sum of Squares	Mean Square	F Value	Prob>F	R-square	η²	ω^2
Model	3	230.05524	76.68508	0.63108	0.61149	0.15919	0.1591867	0
Error	10	1215.13612	121.51361					
Total	13	1445.19137						

Appendix Table 7.9: Statistical analysis for the CT measured RM/TV% in the terminal post-reconstructive follow up point

Descriptive statistics for the CT measured residual biomaterial RM/TV% at second follow up point (terminal endpoint)

Reconstructive gp	N Analysis	Mean	Standard Deviation	SE of Mean
control_end	3	17.85713	11.03012	6.36824
M_end	3	18.45314	4.76078	2.74864
MP_end	4	14.93184	8.84997	4.42499
MVP_end	4	9.79766	7.30445	3.65222

Homogeneity of Variance test: Levene's test (Absolute deviations)

	DF	Sum of Squares	Mean Square	F Value	Prob>F
Model	3	34.59816	11.53272	0.71198	0.56676
Error	10	161.97997	16.198		

At the 0.05 level, the population variances are not significantly different.

One Way ANOVA for the CT measured RM/TV% on terminal endpoint time point

	DF	Sum of Squares	Mean Square	F Value	Prob>F	R-square	η²	ω^2
Model	3	168.20781	56.06927	0.8201	0.5119	0.19745	0.1974512	0
Error	10	683.68788	68.36879					
Total	13	851.89569						

Appendix Table 7.10: Statistical analysis of CT measured RM/TV% between two follow up points

Paired sample t test for each group between the two follow up time points regarding CT measured RM/TV%

		N	Mean	SD	SEM	Median	Hedge’s g		cohen's *d*
RM_contol_post		3	25.67449	13.94549	8.05143	32.9186		0.6665018	0.8163
RM_control_end		3	17.85713	11.03012	6.36824	15.66969			
	Difference	3	7.81736	9.57664	5.52907	3.10152			
	Overall	6	21.76581	12.03285	4.91239	22.74338			
	t Statistic	DF	Prob>|t|						
	1.41386	2	0.29298						

		N	Mean	SD	SEM	Median	Hedge’s g		cohen's *d*
RM_M_post		3	24.54738	10.97626	6.33715	24.18053		0.8005403	0.9805
RM_M_end		3	18.45314	4.76078	2.74864	18.25282			
	Difference	3	6.09424	6.21571	3.58864	5.92771			
	Overall	6	21.50026	8.27039	3.37637	20.78187			
	t Statistic	DF	Prob>|t|						
	1.6982	2	0.23157						

		N	Mean	SD	SEM	Median	Hedge’s g		cohen's *d*
RM_MP_post		4	18.52147	11.23636	5.61818	17.95272		1.1081523	1.2796
RM_MP_end		4	14.93184	8.84997	4.42499	14.29021			
	Difference	4	3.58963	2.80531	1.40266	3.66251			
	Overall	8	16.72665	9.55813	3.37931	14.95668			
	t Statistic	DF	Prob>|t|						
	2.55917	3	0.08327						

		N	Mean	SD	SEM	Median	Hedge’s g		cohen's *d*
RM_MVP_post		4	15.8889	8.29575	4.14788	18.67441		0.8120441	0.9377
RM_MVP_end		4	9.79766	7.30445	3.65222	10.20286			
	Difference	4	6.09124	6.49616	3.24808	3.71093			
	Overall	8	12.84328	7.93482	2.80538	16.01034			
	t Statistic	DF	Prob>|t|						
	1.87533	3	0.15742						

Appendix Table 7.11: Statistical analysis for the biomaterial remodeling rate % across CT time points.

Descriptive statistics of the CT 3D-model comparisons for estimating the remodeling rate % across CT time points

Prefabrication technique	N Analysis	Mean	Standard Deviation	SE of Mean
M	3	0.34636	0.15474	0.08934
MP	4	0.56557	0.14002	0.07001
MVP	4	0.65554	0.22121	0.11061

Homogeneity of Variance test: Levene's test (Absolute deviations)

	DF	Sum of Squares	Mean Square	F Value	Prob>F
Model	2	0.00983	0.00491	0.63461	0.55487
Error	8	0.06193	0.00774		

At the 0.05 level, the population variances are not significantly different.

One Way ANOVA for the 3D model CT measured remodeling rate %

	DF	Sum of Squares	Mean Square	F Value	Prob>F	R-square	η²	ω^2
Model	2	0.16848	0.08424	2.65829	0.13025	0.39925	0.3992512	0.2317
Error	8	0.25351	0.03169					
Total	10	0.42199						

Appendix Table 7.12: Statistical analysis for the µCT measured NB/TV% at terminal endpoint

Descriptive statistics for the µCT measured newly formed bone NB/TV% at terminal endpoint

Reconstructive gp.	N Analysis	Mean	Standard Deviation	SE of Mean
control	3	22.15831	9.74299	5.62512
M	3	17.27836	4.70374	2.71571
MP	4	21.41145	8.33139	4.1657
MVP	4	30.81671	9.33143	4.66572

Homogeneity of Variance test: Levene's test (Absolute deviations)

	DF	Sum of Squares	Mean Square	F Value	Prob>F
Model	3	33.21617	11.07206	1.38698	0.30292
Error	10	79.82838	7.98284		

At the 0.05 level, the population variances are not significantly different.

One Way ANOVA for the µCT measured new bone formation NB/TV%

	DF	Sum of Squares	Mean Square	F Value	Prob>F	R-square	η²	ω^2
Model	3	352.88617	117.62872	1.6719	0.23536	0.33403	0.3340297	0.1259
Error	10	703.56515	70.35652					
Total	13	1056.45132						

Appendix Table 7.13: Statistical analysis for the µCT measured RM/TV% at terminal endpoint

Descriptive statistics for the µCT measured residual biomaterial RM/TV% at terminal endpoint

Reconstructive gp.	N Analysis	Mean	Standard Deviation	SE of Mean
control	3	8.86582	6.9664	4.02205
M	3	12.08487	3.97888	2.29721
MP	4	8.06858	4.7221	2.36105
MVP	4	5.48695	5.98792	2.99396

Homogeneity of Variance test: Levene's test (Absolute deviations)

	DF	Sum of Squares	Mean Square	F Value	Prob>F
Model	3	9.91777	3.30592	0.70774	0.56903
Error	10	46.71128	4.67113		

At the 0.05 level, the population variances are not significantly different.

One Way ANOVA for the µCT measured residual biomaterial RM/TV%

	DF	Sum of Squares	Mean Square	F Value	Prob>F	R-square	η²	ω^2
Model	3	75.74884	25.24961	0.83281	0.50581	0.1999	0.1999	0
Error	10	303.18481	30.31848					
Total	13	378.93364						

Appendix Table 7.14: Statistical analysis of endpoint histological BVs density (vessels/mm²)

Descriptive statistics for the histologically measured blood vessels density (vessels/mm²) terminal endpoint samples

Reconstructive gp.	N Analysis	Mean	Standard Deviation	SE of Mean
control	3	11.82222	1.28985	0.74469
M	3	12.15556	3.21132	1.85406
MP	4	18.5	3.54004	1.77002
MVP	4	31.11667	10.39207	5.19604

Homogeneity of Variance test: Levene's test (Absolute deviations)

	DF	Sum of Squares	Mean Square	F Value	Prob>F
Model	3	87.96409	29.32136	2.35493	0.1334
Error	10	124.51053	12.45105		

At the 0.05 level, the population variances are not significantly different.

One Way ANOVA for the histologically measured vessels/mm² at terminal samples

	DF	Sum of Squares	Mean Square	F Value	Prob>F	R-square	η²	ω^2
Model	3	881.97233	293.99078	7.62555	0.00609	0.69583	0.6958328	0.5867
Error	10	385.5337	38.55337					
Total	13	1267.50603						

Bonferroni means comparisons

Pairs	MeanDiff	SEM	t Value	Prob	Alpha	Sig	LCL	UCL
M control	0.33333	5.06974	0.06575	1	0.05	0	-16.27939	16.946
MP control	6.67778	4.74231	1.40813	1	0.05	0	-8.86201	22.218
MP M	6.34444	4.74231	1.33784	1	0.05	0	-9.19534	21.884
MVP control	19.29444	4.74231	4.06858	0.01353	0.05	1	3.75466	34.834
MVP M	18.96111	4.74231	3.99829	0.01515	0.05	1	3.42133	34.501
MVP MP	12.61667	4.39052	2.87361	0.09939	0.05	0	-1.77038	27.004

Appendix Table 7.15: Statistical analysis for the histologically measured new bone with its related marrow spaces (area %)

Descriptive statistics for the histologically measured new bone with its related marrow spaces (area %)

Reconstructive gp.	N Analysis	Mean	Standard Deviation	SE of Mean
control	3	45.36169	17.80514	10.2798
M	3	32.05304	10.88549	6.28474
MP	4	39.57009	24.16643	12.08322
MVP	4	49.36902	14.65252	7.32626

Homogeneity of Variance test: Levene's test (Absolute deviations)

	DF	Sum of Squares	Mean Square	F Value	Prob>F
Model	3	190.16485	63.38828	0.72929	0.55761
Error	10	869.17334	86.91733		

At the 0.05 level, the population variances are not significantly different.

One Way ANOVA for the histologically measured new bone and marrow areas at terminal samples

	DF	Sum of Squares	Mean Square	F Value	Prob>F	R-square	η²	ω^2
Model	3	571.55654	190.51885	0.58313	0.63949	0.14889	0.1488922	0
Error	10	3267.17163	326.71716					
Total	13	3838.72817						

Appendix Table 7.16: Statistical analysis for the histologically measured residual biomaterial with its related fibrovascular stroma (area %)

Descriptive statistics for the histologically measured residual biomaterial with its related fibrovascular stroma (area %)

Reconstructive gp.	N Analysis	Mean	Standard Deviation	SE of Mean
control	3	54.63831	17.80514	10.2798
M	3	67.94696	10.88549	6.28474
MP	4	60.42991	24.16643	12.08322
MVP	4	50.63098	14.65252	7.32626

Homogeneity of Variance test: Levene's test (Absolute deviations)

	DF	Sum of Squares	Mean Square	F Value	Prob>F
Model	3	190.16485	63.38828	0.72929	0.55761
Error	10	869.17334	86.91733		

At the 0.05 level, the population variances are not significantly different.

One Way ANOVA for the histologically measured residual biomaterial and related fibrovascular stroma at terminal samples

	DF	Sum of Squares	Mean Square	F Value	Prob>F	R-square	η^2	ω^2
Model	3	571.55654	190.51885	0.58313	0.63949	0.14889	0.1488922	0
Error	10	3267.17163	326.71716					
Total	13	3838.72817						

8 References

Bankhead P, Loughrey MB, Fernández JA, Dombrowski Y, McArt DG, Dunne PD, McQuaid S, Gray RT, Murray LJ, Coleman HG et al. 2017. Qupath: Open source software for digital pathology image analysis. Scientific Reports. 7(1):16878.
